# Influencing Factors of Health Status of Clinical Doctors in Tertiary Medical Institutions Based on Structural Equation Modeling

**DOI:** 10.3390/healthcare13233019

**Published:** 2025-11-22

**Authors:** Yangfan Ou, Shanshan Yin, Shuaiyin Chen

**Affiliations:** 1 College of Public Health, Zhengzhou University, Zhengzhou 450001, China; 2 Research Department, Henan Academy of Innovations in Medical Science, Zhengzhou 450003, China

**Keywords:** physical health, social health, mental health, health, structural equation modeling

## Abstract

**Highlights:**

**What are the main findings?**

- Clinicians exhibited significant differences in physical health, mental health, social health, and overall health scores, with mental health scores being the lowest, particularly among younger physicians.
- Age, years of practice, professional title, health status, sleep duration, and exercise duration are significantly associated with the health of clinical physicians.
- Physical health serves as the foundation, influencing both physiological and social well-being, which together are related to a physician’s overall health status.

**What are the implications of the main findings?**

- The health status of clinical physicians, particularly their mental health, warrants special attention.
- Improving physicians’ health requires equal attention to physical, mental, and social well-being—all three are essential.

**Abstract:**

**Objectives**: This study employs structural equation modeling to investigate the health status of clinicians in tertiary medical institutions and its influencing factors. **Methods**: A questionnaire-based survey was conducted on 743 clinicians from five hospitals selected through random sampling, collecting data on their physical health, mental health, social health, and overall health. The analysis examined influencing factors and their interrelationships. **Results**: The results revealed significant differences (*p* < 0.001) in physicians’ scores across physical, mental, social, and overall health, with mental health scoring the lowest (58.85). Factors such as age, years of service, professional title, medical conditions, sleep duration, and exercise duration were significantly associated with clinicians’ health status. Among the 108 physicians (14.5%) diagnosed with medical conditions, mental health scores were significantly lower (*p* < 0.05). Clinicians aged 25~45 years, with 4~10 years of experience, and holding the title of Associate Chief Physician generally scored lower. Physicians with longer sleep duration and exercise duration scored higher in mental and social health. Structural equation modeling analysis revealed that physical health is significantly and positively related to social health (*p* < 0.001). Good physical health is positively related to positive emotions and negatively related to negative emotions. Social support is positively correlated with cognitive function and negatively correlated with negative emotions; meanwhile, enhanced social adaptation shows a significant positive association with both cognitive function and emotional state. **Conclusions**: This study recommends paying particular attention to the health status of clinicians, especially the mental health of young physicians. It is suggested that comprehensive interventions be implemented across the three dimensions of physical, social, and psychological to enhance psychological resilience and perceptions of well-being.

## 1. Introduction

Clinical doctors represent the fundamental element of the medical system, and their health status exerts a direct influence on the quality of medical services and the treatment outcomes of patients. Nevertheless, the working environment of clinical doctors is complex, characterised by high levels of work intensity and considerable pressure. It has been frequently observed that these individuals experience periods of elevated physical and mental strain, which can result in a range of adverse health consequences [1]. In recent years, with the increase in medical demand and the complexity of the medical environment, the health problems of clinical doctors have increasingly attracted attention. A substantial body of research has demonstrated that the physical and mental health status of clinical doctors is suboptimal. This phenomenon has been particularly accentuated during the COVID-19 pandemic, when the prevalence of physical and mental health problems among medical staff has become increasingly evident [2,3].

In recent years, the Job Demands-Resources (JD-R) model has been widely applied to explain the relationship between occupational stress and mental health among healthcare practitioners [4]. This model posits that high job demands, when coupled with insufficient resources, lead to psychological fatigue, emotional exhaustion, and even mental health issues [5]. Social support theory further suggests that support from colleagues, family, and organizations can buffer the negative impact of work stress on mental health, enhancing emotional regulation capacity and occupational well-being [6]. However, the majority of extant research has focused on either physician burnout or specific aspects of physical and mental health [7,8,9,10]. There remains a paucity of studies that systematically integrate the Job Demands-Resources Model with social support theory to explore the underlying mechanisms linking physical, psychological, and social health.

Henan Province is one of the most densely populated regions and is home to one of the country’s largest general hospitals, which is also one of the largest in the world. Clinical physicians in this region face significantly higher workloads [11,12]. Therefore, it is imperative to comprehensively assess the health status of clinical doctors in tertiary medical institutions in Henan Province, along with the factors that influence their well-being. This comprehensive evaluation is crucial for the formulation of targeted health intervention measures aimed at promoting optimal health and well-being among this demographic. The present study constitutes a comprehensive exploration of the health status of clinical doctors in tertiary medical institutions in Henan Province, with a particular focus on the factors that influence this status. This exploration is undertaken through the utilisation of questionnaire-based surveys and statistical analysis, with the objective of providing a scientific basis for the enhancement of the health level of clinical doctors.

This study employed random sampling to conduct a questionnaire-based survey of 743 clinical doctors from five hospitals in tertiary medical institutions in Henan Province. The survey instruments comprised a general situation questionnaire and the Self-Rating Health Measurement Scale (SRHMS) [13], encompassing four dimensions: physical health, mental health, social health, and overall health. A composite score was then calculated based on the scores from these four dimensions. The construct of physical health was further delineated into three subdimensions: physical symptoms, physiological functioning, and activities of daily living. The concept of social health encompasses three distinct dimensions: social support, social connections, and social adaptation. Mental health encompasses positive emotions, negative emotions, and cognitive functioning. The effects of gender, age, years of work experience, educational attainment, marital status, professional title, presence of illness, sleep duration, and exercise habits on clinicians’ health status were analysed using *t*-tests or F-tests. The relationships among physical health, mental health, social health, and overall health were examined by means of exploratory factor analysis (EFA) and structural equation modeling (SEM). The study’s findings may provide a scientific foundation for the development of targeted health intervention measures, with the potential to enhance the overall health status of clinical doctors and, consequently, the quality of medical services.

## 2. Materials and Methods

743 clinical doctors from ten tertiary medical institutions in Henan Province were selected by means of a random sampling method. The inclusion criteria for the study are as follows: Doctors who had been engaged in clinical work for a period of at least six months and had obtained physician registration qualifications. To ensure greater representativeness of the data, we selected hospitals with high provincial recognition and included physicians from various departments in our survey. To minimize questionnaire bias, we employed the established SRHMS and conducted anonymous surveys that avoided sensitive privacy questions. Finally, the questionnaire data was reviewed and any responses deemed to be unreasonable were excluded.

The data were collected using a questionnaire-based survey method. The survey instruments comprised a general situation questionnaire and the Self-Rating Health Measurement Scale (SRHMS) [13]. The SRHMS encompasses four distinct dimensions: physical health, mental health, social health, and overall health, comprising a total of 48 items. These four dimensions can be further subdivided into ten sub-dimensions. The maximum attainable scores for the domains of physical, mental, social, and overall health were determined to be 170, 150, 120, and 40 points, with a maximum total score of 480 points. In order to analyse the relationship between physical health, mental health and social health, we employed EFA to construct a relevant model and validated its reliability using SEM. SEM has been extensively applied in a variety of fields, including psychology, health sciences and social sciences [14]. The model has the capacity to manage relationships among multiple independent and dependent variables, incorporating direct, indirect, and mediating effects, while also accounting for measurement error. This renders it especially well-suited for complex models [14].

The SEM was constructed based on the SRHMS and social support theory. The initial hypothesis proposed a four-dimensional model where physical health influences social health, social health influences psychological health, and all three collectively influence overall health. However, the model’s fit indices were suboptimal. Subsequently, a 10-dimensional measurement model was established, demonstrating excellent model fit. Exploratory factor analysis (EFA) was conducted to validate the dimensions of the 48 scale items, revealing that the factor loadings for each item were at an acceptable level [15]. Prior to constructing the structural equation model, skewness and kurtosis tests were performed on the 48 scale items. Based on the criteria proposed by Kline [16], the data were deemed to follow a normal distribution, enabling the construction of the final structural equation model.

The statistical analysis was conducted utilising SPSS 25.0 software. A descriptive analysis was conducted to describe the current status of the medical staff. The factors related to medical staff, such as gender, age, working years, education level, marital status, professional title, presence or absence of illness, sleep status, and exercise, were compared using independent samples *t*-tests and analysis of variance (ANOVA). The exploratory factor analysis (EFA) and structural equation modeling (SEM) were conducted using AMOS 24.0 software. The significance level was set at α = 0.05.

## 3. Results

### 3.1. General Information

The survey garnered a total of 743 valid questionnaires. The respondents comprised 333 men and 410 women, 177 of whom were single and 566 married; 384 had a bachelor’s degree, 324 had a master’s degree, and 30 had a doctoral degree; 280 were resident physicians, 316 were attending physicians, 119 were associate chief physicians, and 28 were chief physicians. By specialty, the distribution was as follows: 190 in internal medicine, 130 in surgery, 46 in obstetrics and gynecology, 68 in pediatrics, 17 in otorhinolaryngology, 29 in critical care, 27 in emergency medicine, 29 in oncology, 36 in anesthesiology, and 171 in other fields. The mean age of the participants was 34.2 years, and the mean duration of their professional experience was 9.5 years. The mean values for male subjects were found to be 173.8 cm for height and 76.3 kg for weight, while the mean values for female subjects were 162.2 cm for height and 60.3 kg for weight. Of the respondents, 635 (85.5%) reported no diagnosed illnesses, and 108 (14.5%) had one or more diagnosed conditions.

In this study, the *t*-test (Comparison between 2 groups) or F-test (multi-group differential comparison) was employed to examine the association between gender, educational level, and marital status and the physical, mental, social, overall, and total scores of medical workers. The results revealed that these factors did not have a statistically significant effect on the health outcomes of medical workers. However, statistical analysis revealed a statistically significant correlation between age, professional title, sleep duration and exercise duration, and differences in mental health, social health, overall health, and total score. Those aged 45 and over had the highest scores in these four areas. Higher professional titles were associated with higher scores, as were longer sleep and exercise durations. Those with ≥11 years’ work experience had higher social health scores, with no statistically significant differences in other areas. Doctors with a history of diseases had lower scores for mental health, overall health and the total score. There were no statistically significant differences in the physical health and social health scores. The specific data are shown in Table 1.

### 3.2. Health Status of Clinical Doctors in Tertiary Medical Institutions

According to BMI classification, 25 people are underweight (3.4%); 411 people are of a normal weight (55.4%); and 307 people are obese (41.2%). Of those assessed, 29 have hypertension, one has had a stroke, 4 have coronary heart disease, 6 have diabetes, 60 have fatty liver disease, 3 have chronic kidney disease and 43 have dyslipidaemia. The following long-term medication use was reported among the study subjects: antihypertensive drugs (3.5%), antidiabetic drugs (0.8%), lipid-lowering drugs (1.6%), uric acid-lowering drugs (0.1%), antiarrhythmic drugs (0.1%), antipyretic and analgesic drugs (0.4%), corticosteroid drugs (0.1%), oestrogenic drugs (0.3%), sedative-hypnotic drugs (0.4%), antithyroid drugs (0.4%), hepatoprotective drugs (0.3%), and antidepressant drugs (0.1%). Among the study subjects, 108 individuals (14.5%) had undergone surgical treatment due to illness, with the top three categories being gastrointestinal surgery, gynaecological surgery and neck/thyroid surgery. The specific data are shown in Table 2.

Table 3 shows health scale scores for each dimension of the health scale across all study participants. There are statistically significant differences (*p* < 0.001) in physical health, mental health, social health, overall health and total scores among clinical doctors. The lowest score is for mental health at 88.27 ± 24.32, equivalent to 58.85% of the maximum score. The highest score is for physical health at 127.46 ± 21.32, equivalent to 74.98% of the total score.

### 3.3. Reliability Analysis of the Questionnaire and Results of CFA

The reliability of the questionnaire was analysed using Cronbach’s Alpha reliability test. The value of Cronbach’s Alpha ranges from 0 to 1, with higher values indicating higher reliability. In general, a coefficient below 0.6 is considered unreliable, suggesting that the questionnaire should be redesigned or that the data should be re-collected for analysis. It is widely accepted within the field that a reliability coefficient between 0.6 and 0.7 is considered acceptable, between 0.7 and 0.8 is considered fairly reliable, between 0.8 and 0.9 is considered very reliable, and between 0.9 and 1 is considered highly reliable. In this analysis, the reliability coefficients for physical health, mental health, social health, overall health, and the entire scale were 0.863, 0.905, 0.908, 0.847, and 0.944, respectively. Given that all the coefficients for each dimension and the overall scale are above 0.8, it can be deduced that the questionnaire design has a high level of credibility. The specific data are shown in Table 4.

The evaluation indicators of the Confirmatory Factor Analysis (CFA) model generally include the ratio of chi-square to degrees of freedom (CMIN/DF), the root mean square error of approximation (RMSEA), the Tucker–Lewis index, the comparative fit index (CFI), the incremental fit index (IFI), and the goodness-of-fit index (GFI), among others [17]. The evaluation results of the model’s fit showed that the CMIN/DF was 3.314 and the RMSEA was 0.056, which, although slightly higher than 0.05, was still within the acceptable range. The final results indicated that the model exhibited a strong degree of fit with the data. Furthermore, the factor loadings of all items exceeded 0.5 (*p* < 0.001), suggesting adequate internal consistency within each dimension. The specific results are displayed in the following Table 5.

### 3.4. Structural Equation Model (SEM) Analysis

**Figure 1:**
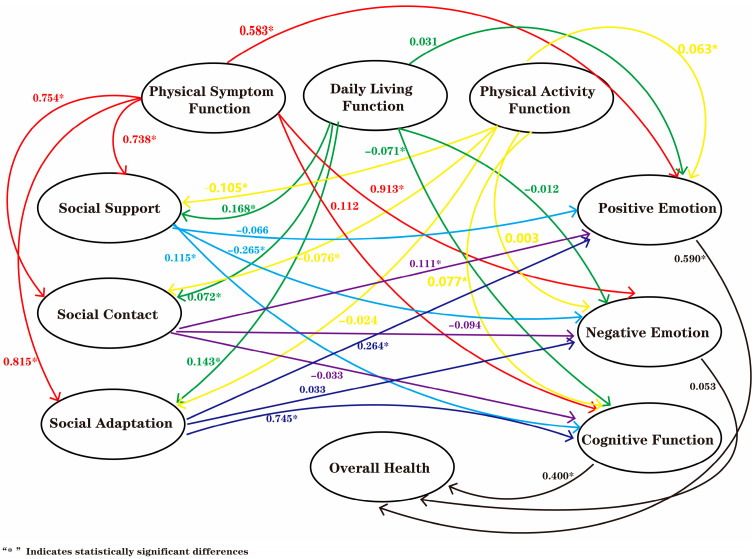
Structural Equation Model of Clinicians’ Physical Health, Mental Health, Social Health, and Overall Health.

The final structural equation model diagram is shown in Figure 1. The model’s CMIN/DF value is 4.205, the RMSEA value is 0.066, and the TLI, CFI, IFI, GFI, and AGFI values are 0.836, 0.847, 0.848, 0.803, and 0.779, respectively. The following Table 6 illustrates the aforementioned path relationships: The impact of physical symptom function on social support, social contact, and social adaptation (social health) is significant and positive, with path coefficients of 0.738, 0.754, and 0.815 (*p* < 0.05), respectively. Furthermore, daily living function has a significant positive impact on social support, social contact, and social adaptation (social health), with path coefficients of 0.168, 0.072, and 0.143 (*p* < 0.05), respectively. Conversely, physical activity function has a detrimental effect on social support and social contact, with path coefficients of −0.105 and −0.076, respectively. The impact of physical symptom function on positive and negative emotions has been demonstrated to be positive (path coefficients = 0.583 and 0.913, respectively). Furthermore, the impact of daily living function on negative emotions and cognitive function has been shown to be slightly negative (path coefficients = −0.012 and −0.071, respectively). In addition, physical activity function has been evidenced to have a positive impact on positive emotions and cognitive function (path coefficients = 0.063 and 0.077, respectively). Moreover, social adaptation has been demonstrated to directly affect cognitive function and positive emotions (path coefficients = 0.745 and 0.264, respectively). In addition, social contact has been shown to directly affect positive emotions (path coefficient = 0.111). Finally, social support has been evidenced to directly affect cognitive function and negative emotions (path coefficients = 0.115 and −0.265, respectively). The present study has demonstrated that cognitive function exerts a direct effect on overall health (path coefficient = 0.400) and that positive emotions have a direct effect on overall health (path coefficient = 0.590).

## 4. Discussion

### 4.1. Factors Associated with the Health of Clinical Doctors

It is evident that clinical doctors encounter elevated workloads and considerable levels of pressure in their professional activities [18,19]. This is further compounded by the intricate nature of their work environment, which engenders challenges to their physical and mental well-being [18,19]. Table 2 reveals that the majority of doctors encounter health concerns to various extents. The study comprehensively analysed the relationship between the health of clinical doctors and a number of factors, including gender, age, working years, education level, marital status, professional title, presence of illness, sleep status, and exercise habits. These factors were considered across four distinct dimensions: physical health, mental health, social health and overall health. The study investigated the potential factors influencing the health of clinical doctors, utilising a structural equation model to analyse the interrelationships among physical, mental, and social health.

As illustrated in Table 3, within the domains of physical, mental, and social health in clinical physicians, physical health scores were found to be comparatively high, while mental health scores were found to be lower. The differences observed were found to be statistically significant. Research has indicated that medical professionals are more prone to psychological health issues [20,21]. As demonstrated in the relevant literature, there is a significant correlation between psychological health and professional lives [22,23]. Table 2 indicates that gender, educational attainment, and marital status are not significantly associated with clinicians’ physical health, mental health, social health, overall health, and total health scores. Several studies have indicated that women experience elevated levels of stress in the physical and mental domains [24,25]. Conversely, another study has suggested that men encounter heightened pressure as a result of familial economic factors [26]. The relationships between the health status of clinical doctors and their gender, level of education, and marital status are not straightforward. These factors are more likely to interact with other sociodemographic characteristics or work environment factors to jointly influence the health status of clinical doctors.

In relation to the associations between working years and clinicians’ health, physicians with 4 to 10 years of experience demonstrate the lowest social health scores. During this phase, clinicians are confronted with an escalating degree of intricacy in social dynamics, whilst concomitantly focusing on clinical duties. This is coupled with pressures stemming from doctor-patient relationships and professional title promotions. Furthermore, professional titles have been demonstrated to influence physicians’ health scores, with attending physicians exhibiting the lowest social health scores. This finding is consistent with the pattern of lower social health scores among clinicians with 4 to 10 years of experience. Furthermore, a study conducted during the COVID-19 pandemic also demonstrated that healthcare professionals in subordinate roles are more prone to psychological distress due to their comparatively limited professional experience and paucity of accumulated clinical knowledge [3]. The medical history of clinical physicians has been demonstrated to exert an influence solely on mental health and overall health, with no effect on physical health scores. In principle, a medical diagnosis should have a tangible impact on an individual’s physical health status. However, given that the study subjects were working clinical doctors who have undergone systematic medical education and training and possess higher health literacy and self-health management capabilities, they usually pay more attention to healthy behaviours and lifestyles [27]. These healthy behaviours may help mitigate the adverse effects of diseases on physical health, with the effect being mainly on mental health status. Regarding the associations between sleep duration and average weekly exercise duration with health outcomes, it was found that sleep duration and exercise duration are positively correlated with scores for physical health, social health, and mental health. International research has found that adequate sleep duration [28,29] and physical activity [30,31] play a significant role in alleviating occupational burnout and improving the mental health of healthcare workers. This finding is consistent with the results of the present study. Another study demonstrated that medical workers exhibit a lower level of physical exercise, with 70.98% of medical workers demonstrating an absence of such activity [32]. This has been shown to result in a sub-healthy and fatigued state.

### 4.2. The Relationship Between Physical Health and Social and Mental Health

The results of the structural equation model reveal that physical symptom function exerts a significant positive influence on three dimensions of social health: social support, social contact, and social adaptation. This indicates that the enhancement of physical symptom function can significantly strengthen clinicians’ sense of social support, frequency of social contact, and capacity for social adaptation. This finding aligns with the core tenets of social support theory, which posits that optimal physical health enhances an individual’s social functioning [33,34]. A study conducted in Israel has indicated that a positive physical environment can significantly enhance physicians’ sense of social support, thereby reducing the occurrence of occupational burnout [35]. While the three dimensions of social health are positively associated with daily living function, the path coefficients are relatively weak, indicating a lesser role in promoting social health. Furthermore, a negative correlation was identified between physical activity and both social support and social contact. This finding suggests that excessive physical activity among clinicians may reduce opportunities for interpersonal interaction or diminish perceptions of social support. The result also indicated that good physical health is positively correlated with clinicians’ emotional well-being, enhancing positive emotions while reducing the occurrence of negative emotions. Some studies have found that individuals who maintain good physical health are better equipped to enhance positive emotions and regulate negative emotions through cognitive reappraisal [36,37].

### 4.3. The Relationship Between Social Health and Mental Health

The result of SEM revealed that social support could enhance cognitive function and alleviate negative emotions. A British interview-based study found that most of the negative emotions experienced by general practitioners stem from a lack of social support [38]. It has been posited by certain studies that social support has the capacity to substantially alleviate symptoms of depression and anxiety, thereby indirectly enhancing cognitive function [39,40]. Furthermore, social adaptation exerts a direct influence on cognitive function and positive emotions, suggesting that enhancing social adaptation capabilities can substantially improve clinical doctors’ cognitive function and emotional well-being. This finding lends further support to the social support theory, which posits that a robust social support system can serve as a cushion against the deleterious effects of work stress on mental well-being.

### 4.4. The Relationship Between Mental Health and Overall Health

A multi-country serial cross-sectional study found that depression was positively correlated with COVID-19 incidence and mortality rates, indicating that mental health severely jeopardized physicians’ health status [41]. This model reveals that cognitive function and positive emotions have a significant direct association with overall health, while negative emotions show no significant association. Research indicates that positive emotions can enhance an individual’s psychological resilience, helping them better cope with stress and challenges, and exerting a significant protective effect on their perception of health [42]. The possession of good cognitive function by individuals enables them to assess their health status with greater clarity and also to enhance their ability to cope with health issues [43]. Despite the theoretical possibility of negative emotions having a detrimental effect on overall health, they did not have a significant effect in the actual model. The impact of negative emotions may be overshadowed by the positive effects of positive emotions and cognitive function. In sophisticated psychological and health models, the impact of negative emotions may only become apparent over a protracted period or in more complex models [44].

## 5. Limitations

While our study provides valuable insights into the health status of clinical doctors in Henan Province, it is important to recognize its limitations. First, the cross-sectional design only allows for the observation of statistical associations between variables, without establishing temporal sequences or causal relationships. Second, the study was conducted exclusively in Henan Province, limiting the generalizability of the findings. Future research should consider expanding to multiple provinces to enhance the external validity of findings. Finally, this study did not incorporate background variables such as workload, working conditions, or institutional characteristics. The absence of these variables may limit the comprehensive interpretation of results. Future research should account for these factors to provide more accurate conclusions.

## 6. Conclusions

Working years, professional title, presence of illness, sleep status, and exercise habits are the main factors influencing the health status of clinicians in Henan Province. Physical health, social health, and mental health are interrelated, collectively impacting an individual’s overall health level. Physical health is associated with higher levels of social support and positive emotions, and lower levels of negative emotions. Social health is related to better mental health through mechanisms involving social support and social adaptation, which in turn are associated with improved cognitive function and emotional states. At the mental health level, cognitive function and positive emotions are significantly associated with overall health, whereas negative emotions have a negligible impact. Therefore, elevating an individual’s overall health requires comprehensive intervention across physiological, social, and psychological dimensions to strengthen psychological resilience and health perception.

## Figures and Tables

**Table 1:** Clinical Doctors’ Health Self-assessment and Analysis of Influencing Factors.

Variable	n	Physical Health (170)	Mental Health (150)	Social Health (120)	Overall Health (40)	Total Score (480)
Gender						
Male	333	127.64 ± 22.51	88.38 ± 23.05	80.20 ± 79.46	26.62 ± 6.94	322.92 ± 53.69
Female	410	127.31 ± 20.32	88.20 ± 25.30	79.46 ± 18.72	26.82 ± 7.30	321.77 ± 57.60
Age						
<25	25	124.92 ± 26.85	89.48 ± 22.37 *	81.12 ± 18.13 *	28.72 ± 5.66 *	324.24 ± 51.18 *
<45	636	127.70 ± 20.96	86.95 ± 24.00	78.53 ± 18.27	26.30 ± 7.20	319.49 ± 55.65
≥45	82	126.34 ± 22.43	98.20 ± 25.29	89.50 ± 16.99	29.39 ± 6.34	343.43 ± 54.73
Working Years						
<5	212	128.20 ± 21.90	88.85 ± 23.94	80.89 ± 17.15 *	27.54 ± 6.64	325.50 ± 53.31
<11	300	127.74 ± 20.38	86.54 ± 25.44	77.49 ± 19.42	26.09 ± 7.70	317.86 ± 59.11
≥11	231	126.40 ± 22.01	90.00 ± 23.11	81.90 ± 17.99	26.80 ± 6.76	325.09 ± 53.57
Education Level						
Bachelor	384	126.30 ± 23.11	88.92 ± 23.71	80.84 ± 18.20	27.09 ± 7.04	323.16 ± 50.26
Master	324	129.00 ± 18.26	87.40 ± 24.82	78.94 ± 18.21	26.19 ± 7.17	321.53 ± 54.50
Doctorate	30	124.27 ± 28.18	90.20 ± 27.06	78.60 ± 23.41	27.67 ± 7.99	320.73 ± 68.46
Others	5	135.60 ± 4.72	83.80 ± 25.80	67.60 ± 15.19	27.00 ± 4.90	314.00 ± 37.35
Marital Status						
Unmarried	177	127.28 ± 22.25	88.20 ± 25.95	80.76 ± 18.90	27.24 ± 7.52	323.49 ± 60.44
Married	566	127.51 ± 21.04	88.30 ± 23.81	79.54 ± 18.29	26.56 ± 7.00	321.91 ± 54.38
Professional Title						
Resident physician	280	128.26 ± 21.95	89.16 ± 25.31 *	80.84 ± 18.82 *	27.64 ± 7.22 *	325.90 ± 58.69 *
Attending Physician	316	126.34 ± 21.62	85.03 ± 23.27	77.21 ± 18.01	25.45 ± 7.112	333.33 ± 51.95
Associate Chief Physician	119	129.37 ± 18.72	93.50 ± 23.39	82.80 ± 18.14	27.66 ± 6.62	305.6 ± 46.68
Chief Physician	28	123.82 ± 21.78	93.75 ± 25.38	86.71 ± 16.66	28.11 ± 6.61	332.39 ± 57.38
Disease						
With Disease	108	127.02 ± 18.23	82.11 ± 23.73 *	76.97 ± 17.36	23.63 ± 7.19 *	309.73 ± 52.40 *
Without Disease	635	127.53 ± 21.81	89.32 ± 24.28	80.33 ± 18.58	27.25 ± 7.00	324.42 ± 56.17
Sleep Duration						
≤5 h	28	120.32 ± 22.58	75.79 ± 26.87 *	70.71 ± 21.64 *	20.93 ± 9.51 *	287.75 ± 65.71 *
<7 h	545	127.79 ± 20.53	86.52 ± 24.14	79.09 ± 18.19	26.35 ± 6.94	319.74 ± 54.83
≥7 h	170	127.56 ± 23.42	95.95 ± 22.61	83.72 ± 17.91	28.89 ± 6.57	336.13 ± 54.00
Exercise Duration						
0 h	139	126.91 ± 18.51	79.96 ± 25.14 *	73.33 ± 18.42 *	24.12 ± 7.91 *	304.32 ± 56.33 *
<2 h	427	127.76 ± 19.67	86.63 ± 23.11	78.76 ± 17.96	26.27 ± 6.85	319.42 ± 54.08
<5 h	130	126.05 ± 26.70	97.65 ± 23.79	87.19 ± 17.80	29.51 ± 6.25	340.39 ± 55.09
≥5 h	47	130.19 ± 26.57	101.89 ± 21.00	88.40 ± 14.84	30.89 ± 5.17	351.38 ± 48.52

Note: The * indicates that the difference is statistically significant after a *t*-test (or F-test).

**Table 2:** Illness History of Clinical Doctors in Tertiary Medical Institutions.

Disease	n	(%)	Average Age at Diagnosis
Hypertension	29	3.9	36.4
Stroke	1	0.1	29.0
Coronary Heart Disease	4	0.5	45.0
Diabetes	6	0.8	48.8
Fatty Liver	60	8.1	33.9
Chronic Kidney Disease	3	0.4	30.3
Abnormal Lipid Levels	43	5.8	36.2

**Table 3:** Overall Scores of Various Dimensions of the SRHMS.

Variable	Mean	SD	Min	Max	Percentage	*F*	*p*
Physical Health (170)	127.46	21.32	29.00	168.00	74.98	177.89	<0.001
Mental Health (150)	88.27	24.32	12.00	150.00	58.85		
Social Health (120)	79.83	18.43	16.00	120.00	66.53		
Overall Health (40)	26.73	7.13	3.00	40.00	66.83		
Total Score (480)	322.29	55.85	144.00	477.00	67.14		

**Table 4:** Results of Questionnaire Reliability Analysis.

Variable	Cronbach’s Alpha	Number of Items
Physical Health	0.863	17
Mental Health	0.905	15
Social Health	0.908	12
Overall Health	0.847	4
Overall reliability	0.944	48

**Table 5:** Fit Indices for CFA Model Evaluation.

Fit Index	Ideal Standard	General Standard	Result
CMIN/DF	1–3	3–5	3.314
RMSEA	<0.05	<0.08	0.056
TLI	>0.9	>0.8	0.903
CFI	>0.9	>0.8	0.893
IFI	>0.9	>0.8	0.903
GFI	>0.9	>0.8	0.842
AGFI	>0.9	>0.8	0.818

**Table 6:** Path Relationships of SEM for Self-assessment Health Scale.

Path Relationship			Path Coefficient	*p*
Social Support	←	Physical Symptom Function	0.738	<0.001
Social Contact	←	Physical Symptom Function	0.754	<0.001
Social Adaptation	←	Physical Symptom Function	0.815	<0.001
Social Support	←	Daily Living Function	0.168	<0.001
Social Contact	←	Daily Living Function	0.072	0.025
Social Adaptation	←	Daily Living Function	0.143	<0.001
Social Support	←	Physical Activity Function	−0.105	0.004
Social Contac	←	Physical Activity Function	−0.076	0.018
Social Adaptation	←	Physical Activity Function	−0.024	0.43
Positive Emotion	←	Physical Symptom Function	0.583	<0.001
Negative Emotion	←	Physical Symptom Function	0.913	<0.001
Cognitive Function	←	Physical Symptom Function	0.112	0.289
Positive Emotion	←	Daily Living Function	0.031	0.319
Negative Emotion	←	Daily Living Function	−0.012	0.788
Cognitive Function	←	Daily Living Function	−0.071	0.028
Positive Emotion	←	Physical Activity Function	0.063	0.022
Negative Emotion	←	Physical Activity Function	0.003	0.936
Cognitive Function	←	Physical Activity Function	0.077	0.008
Cognitive Function	←	Social Adaptation	0.745	<0.001
Negative Emotion	←	Social Adaptation	0.033	0.713
Positive Emotion	←	Social Adaptation	0.264	<0.001
Cognitive Function	←	Social Contact	−0.033	0.513
Negative Emotion	←	Social Contact	−0.094	0.165
Positive Emotion	←	Social Contact	0.111	0.025
Cognitive Function	←	Social Support	0.115	0.044
Negative Emotion	←	Social Support	−0.265	<0.001
Positive Emotion	←	Social Support	−0.066	0.235
Overall Health	←	Cognitive Function	0.4	<0.001
Overall Health	←	Negative Emotion	0.053	0.065
Overall Health	←	Positive Emotion	0.59	<0.001

## Data Availability

This data survey examines the basic information and family circumstances of clinical physicians in Henan Province. To protect the privacy of individuals, it is intended solely for use within this research and cannot be made publicly available.
